# Expression Profiles Reveal Parallel Evolution of Epistatic Interactions Involving the CRP Regulon in Escherichia coli

**DOI:** 10.1371/journal.pgen.0040035

**Published:** 2008-02-15

**Authors:** Tim F Cooper, Susanna K Remold, Richard E Lenski, Dominique Schneider

**Affiliations:** 1 Department of Biology and Biochemistry, University of Houston, Houston, Texas, United States of America; 2 Department of Biology, University of Louisville, Louisville, Kentucky, United States of America; 3 Department of Microbiology and Molecular Genetics, Michigan State University, East Lansing, Michigan, United States of America; 4 Laboratoire Adaptation et Pathogénie des Micro-organismes, Université Joseph Fourier Grenoble 1, Grenoble, France; 5 CNRS UMR 5163, Grenoble, France; University of Toronto, Canada

## Abstract

The extent and nature of epistatic interactions between mutations are issues of fundamental importance in evolutionary biology. However, they are difficult to study and their influence on adaptation remains poorly understood. Here, we use a systems-level approach to examine epistatic interactions that arose during the evolution of Escherichia coli in a defined environment. We used expression arrays to compare the effect on global patterns of gene expression of deleting a central regulatory gene, *crp*. Effects were measured in two lineages that had independently evolved for 20,000 generations and in their common ancestor. We found that deleting *crp* had a much more dramatic effect on the expression profile of the two evolved lines than on the ancestor. Because the sequence of the *crp* gene was unchanged during evolution, these differences indicate epistatic interactions between *crp* and mutations at other loci that accumulated during evolution. Moreover, a striking degree of parallelism was observed between the two independently evolved lines; 115 genes that were not *crp*-dependent in the ancestor became dependent on *crp* in both evolved lines. An analysis of changes in *crp* dependence of well-characterized regulons identified a number of regulatory genes as candidates for harboring beneficial mutations that could account for these parallel expression changes. Mutations within three of these genes have previously been found and shown to contribute to fitness. Overall, these findings indicate that epistasis has been important in the adaptive evolution of these lines, and they provide new insight into the types of genetic changes through which epistasis can evolve. More generally, we demonstrate that expression profiles can be profitably used to investigate epistatic interactions.

## Author Summary

The effect of a genetic mutation can depend on the genotype of the organism in which it occurs. For example, a mutation that is beneficial in one genetic background might be neutral or even deleterious in another. The interactions between genes that cause this dependence—known as epistasis—play an important role in many evolutionary theories. However, they are difficult to study and remain poorly understood. We used a novel approach to examine the evolution of interactions arising between a key regulatory gene, *crp*, and mutations that occurred during the adaptation of a bacterium, Escherichia coli, to a laboratory environment. To do this, we measured the effect of deleting *crp* on the expression of all genes in the organism, providing a sensitive measure to identify new interactions involving this gene. We found that deleting *crp* had a dramatic and parallel effect on gene expression in two independently evolved populations, but much less effect in their ancestor. An analysis of these changes identified a number of regulatory genes as candidates for harboring beneficial mutations that could account for the parallel changes. These findings indicate that epistasis has played an important role in the evolution of these populations, and they provide insight into the types of genetic changes through which epistasis can evolve.

## Introduction

Epistatic interactions are revealed when the contribution of a mutation to an organism's phenotype depends on the genetic background in which it occurs. Epistasis plays an important role in many evolutionary theories, including those seeking to explain speciation [1], the evolution of sex [2–5], and adaptation [6–10]. In practice, however, epistatic interactions are usually difficult to study and their role in the evolution of organisms therefore remains unclear.

Approaches based on quantitative-trait loci have been increasingly used to study epistasis [11–15]. Although these techniques have the advantage of being quite general, they suffer from some shortcomings including low statistical power, difficulty in detecting some types of epistatic interactions, and inapplicability to non-recombining organisms [11,16]. Recently, systems-level approaches have been developed that avoid some of these problems [17,18]. These approaches typically assess epistatic interactions by comparing the individual and pair-wise effects of large numbers of defined mutations, allowing the outline of functional biological modules and biochemical pathways to be determined [19–23]. To date, however, most systems-level studies have focused on deletion and other knockout mutations, and it is not clear whether findings of widespread epistasis are representative of mutations involved in adaptive evolution.

Bacteria and viruses are ideal organisms with which to conduct controlled evolution experiments owing to their ease of culture and short generation times, as well as the capacity to store them in a non-evolving state from which they can later be revived to allow direct comparisons between ancestral and derived states (reviewed in [24]). These experiments have allowed examination of many aspects of adaptation, including a variety of studies on the nature and extent of epistatic interactions that affect evolution [25–33]. One aspect in common to most of these studies is that they assess epistasis through the effects of mutations on fitness or some related high-level phenotype. However, at the biochemical level, it is easy to imagine that interactions might combine to create a non-linear mapping to fitness [34]. Moreover, inference of epistatic interactions from fitness alone does not usually give any insight into their underlying genetic and physiological causes.

In this study, we combine a systems-level approach with a model experimental system to examine epistatic interactions that arose during the independent adaptation of two lines of E. coli to a glucose-limited minimal medium during 20,000 generations [35,36]. Specifically, we ask whether epistatic interactions occur between a key global regulatory gene, *crp*, and mutations that arose during the course of the experimental evolution; and, if so, to what extent these interactions evolved in parallel in the two lines. Parallel changes in independently evolving lines are of special interest because they are often characteristic of adaptive evolution, and thus call attention to those genes and phenotypic traits that have been important targets of selection [37–43].

We chose to examine interactions at the level of transcription and involving *crp* for several interrelated reasons. First, CRP (cAMP receptor protein, previously known as catabolite activator protein (CAP)) is a key hub in the E. coli transcriptional network. In fact, CRP is involved in more than 200 direct regulatory interactions [44–47], which makes it a good candidate to have evolved interactions with mutations fixed during the evolution experiment. Consistent with this possibility, the evolved lines underwent substantial changes in their carbon-utilization profiles, and CRP is known to play a key role in regulating use of carbon sources in response to environmental signals including glucose concentration [48,49]. The *crp* gene has not itself acquired any new mutations during the evolution experiment, but new epistatic interactions may have arisen between *crp* and mutations that evolved in other genes [49]. Second, we have shown previously that transcription profiles changed during this evolution experiment and that at least some of these changes were caused by evolved differences in regulatory interactions [37]. Finally, mutations in regulatory genes known to depend on *crp* have been identified in the evolving populations [40,50].

In order to detect epistatic interactions, we compared the effect of deleting *crp* on the expression profiles of the ancestral and two evolved strains. Thus, we focused attention on evolutionary changes that affected the CRP regulon, even though the sequence of the *crp* gene itself did not change at all during the evolution experiment [37]. Expression profiles represent high-resolution phenotypes consisting of many low-level traits, thus allowing detailed comparisons of the effects of deleting *crp*. If the set of genes with which *crp* interacts remained constant during the evolution experiment, then its deletion should cause the same expression changes in the ancestral and evolved strains. However, if new epistatic interactions evolved, or if the strengths of existing interactions have changed, then we expect the *crp* deletion to have different effects in the evolved lines from those in the ancestor. Also, we can address the extent of parallelism in evolved changes in epistatic interactions by comparing the expression profiles of the *crp* mutants in the two independently evolved lines.

## Results

### Effect on Growth Rate of Deleting *crp* in Evolved and Ancestral Strains

Deleting the *crp* gene caused significant reductions in the growth rate of the Ara+1 and Ara-1 evolved strains and in their ancestor (paired *t*-tests, all *p* < 0.001). The growth rate of the *crp* ^+^ genotype was 1.35 (SEM ± 0.03) times higher than that of the isogenic *crp* ^−^ deletion mutant in the ancestral genetic background. The corresponding growth rate differentials of the *crp* ^+^ and *crp* ^−^ genotypes were 5.92 fold (SEM ± 0.13) and 13.01 fold (SEM ± 0.57) in the evolved Ara+1 and Ara-1 strains, respectively. In both cases, these differences represent significantly more deleterious effects of the *crp* deletion in the evolved strains than in the ancestor (*t*-tests, both *p* < 0.0001). These results are consistent with the possibility that interactions between *crp* and genes elsewhere in the genome were substantially altered during the evolution experiment*.*

### Changes to the CRP Regulon during the Evolution Experiment

We can define the CRP regulon as the set of genes whose expression levels depend on *crp*. This set is defined operationally as all those genes that show significant differences in standardized expression levels depending on whether the wild-type *crp* ^+^ allele or the *crp* ^–^ deletion allele is present. We do not attempt to distinguish whether the regulation by CRP is direct or indirect. The questions we aim to address are whether the CRP regulon changed during the evolution experiment and, if so, whether it changed in parallel ways in the two independently derived lines. To assist our interpretation of this large and complex dataset, we further define the *core* regulon as those genes whose expression depended on *crp* in *all* three strains, and the *meta*-regulon as those genes dependent on *crp* in *any* of the three strains.

The Venn diagram in Figure 1 shows all the CRP regulons inferred from analyses of gene-expression data in the three strains. The small circle containing regions D, E, F and G represents the regulon inferred from the dependence of gene expression on *crp* in the ancestral strain. A total of 171 genes depended on *crp* in this strain, which is consistent with results of a previous study using a K-12 strain of E. coli [45]. The union of all seven regions, A-G, comprises the meta-regulon inferred by combining data from the ancestral and both evolved strains. The much smaller core regulon is represented by region E only, which is the intersection of the regulons independently inferred across all three strains. Scatter plots highlighting the relationships between *crp*-dependent genes across the three strains are shown in Figure 2.

**Figure 1:**
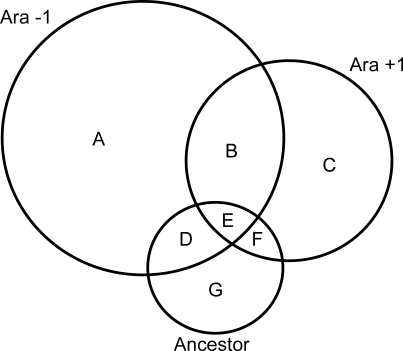
Venn Diagram Showing the Relationship among the CRP Regulons Inferred for Three Genetic Backgrounds Based on Their Whole-Genome Transcription Profiles Ara-1 and Ara+1 are two lineages independently derived from the Ancestor by evolution for 20,000 generations in glucose-limited minimal medium. The union of all three circles defines the *crp* meta-regulon, while their intersection (region E) defines the ‘core' regulon. Those genes in the intersection between the two evolved lines (region B) have independently evolved dependence on *crp*. The size of each circle is proportional to the number of genes in the corresponding inferred CRP regulon. The numbers of genes in each section of the diagram are as follows: A, 569; B, 117; C, 232; D, 30; E, 25; F, 18; G, 98. See text for further details.

**Figure 2:**
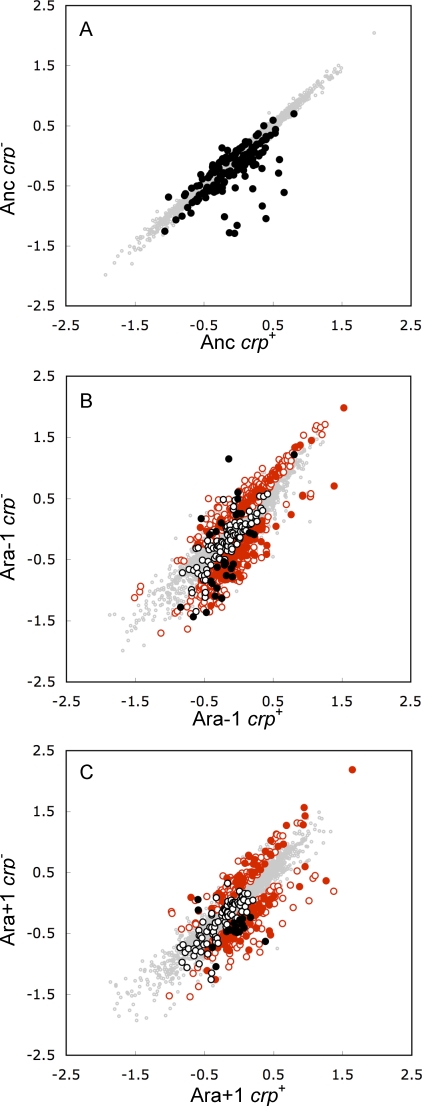
Scatter Plots Showing Effects of the *crp* Deletion on Expression Profiles of the Ancestor and Two Evolved Lines All axes are log_10_-transformed standardized expression levels. Points falling above (below) the diagonal have higher (lower) expression in the absence of *crp*. (A) Comparison of ancestor and its *crp* ^−^ derivative. (B) Comparison of evolved Ara-1 strain and its *crp* ^−^ derivative. (C) Comparison of evolved Ara+1 strain and its *crp* ^−^ derivative. Genes whose expression was significantly (*p* < 0.05) affected by the *crp* deletion are highlighted as follows. (A) Solid black symbols, dependent on *crp* in the ancestor (Figure 1, regions D, E, F, and G). (B,C) Solid black symbols, *crp*-dependent in the ancestor and corresponding evolved line (Figure 1, regions D and E for Ara-1, regions E and F for Ara+1); hollow black symbols, *crp*-dependent in ancestor only (Figure 1, regions F and G for Ara-1, regions D and G for Ara+1); solid red symbols, newly *crp*-dependent in both evolved lines (Figure 1, region B); hollow red symbols, newly *crp*-dependent in only one evolved line (Figure 1, region A for Ara-1, region C for Ara+1). Genes that were not *crp*-dependent in any of the three backgrounds are shown as gray dots in all three panels.

Several features of this analysis are striking. First, the *crp* meta-regulon is very large, comprising some 1,089 genes (about 25% of all genes). Second, the ancestral and core regulons are both much smaller, containing only 171 and 25 genes, respectively. Therefore, the evolved strains exhibit greatly expanded CRP regulons, compared to the ancestor. Third, and more subtly, there is much more overlap in the CRP regulons for the two evolved strains than for either evolved strain and its ancestor. The intersection for the two evolved lines is about 14% [= (B + E)/(A + B + C + D + E + F)], while the intersection for each evolved line and the ancestor is only 7% or 8% for Ara-1 and Ara+1, respectively. Of course, the Venn diagram must be interpreted cautiously because each underlying datum – a test of the difference in gene expression between *crp* ^+^ and *crp* ^−^ genotypes – is subject to statistical uncertainty. In the analyses that follow, we demonstrate that these striking features are real, and not mere artifacts, by performing a series of more constrained and rigorous statistical tests.

### Statistical Support for Global Changes to the CRP Regulon

Statistical analyses must, in general, consider two types of errors: false positives and false negatives. False positives arise when a test yields a nominally significant result, while in fact there is no real difference between the comparison groups. False negatives occur when there is a difference between comparison groups, but a statistical test indicates the observed difference is not significantly greater than expected by chance alone. False positives are especially important to consider when one performs numerous tests on large datasets, as in this study, where we compare expression levels between *crp* ^+^ and *crp* ^−^ genotypes for several thousand genes in each of three genetic backgrounds. We now employ two different criteria to address whether the results summarized above and in Figure 1 could reflect false positives. Importantly, these tests do not focus on the narrow inferences of whether each particular gene is part of a CRP regulon as defined above, but instead these tests focus on the broad inference of whether the expression data, viewed as a whole, support the summary results stated above.

The first test relies on a simple comparison between the total number of genes we identified as belonging to the CRP regulon and the number of false positives that might be included in the CRP regulon given the nominal significance level. That significance level, which was 5% in our tests, describes the likelihood of a false positive arising by chance. Given that there were 4290 genes in the analysis, one could expect 4290 × (1 − (1 − 0.05)^3^)) ≈ 612 false positives to be distributed approximately equally among the three genetic backgrounds that comprise the entire *crp* meta-regulon, where the exponent reflects the fact that independent tests were performed in three strains**.** This number is substantially less than the 1089 genes identified as belonging to the *crp* meta-regulon, and the discrepancy between observed and expected proportions is highly significant based on a binomial test (*p* < 0.0001). Therefore, while some false positives are undoubtedly included in the meta-regulon, the expression levels for many genes thus identified do indeed depend on whether the *crp* ^+^ or *crp* ^−^ allele is present in one or more of the three strains.

The second set of tests asks whether there is significant concordance across the strains in the directionality of differences in gene expression depending on which *crp* allele is present. Four such sign tests are possible; one test involves all three strains and the other three tests include the various pairs of strains (Table 1). Region E is the narrowly defined *crp* core regulon, which includes only the 25 genes whose expression level was significantly affected by the *crp* allele in all three strains. If this core regulon was a statistical anomaly, whereby measurement noise led to false positives, then we would expect half of the comparisons to show higher expression in the *crp* ^+^ genotype than in the *crp* ^−^ genotype, and half to exhibit the opposite pattern. Given three strains, we expect only 0.5^3^ × 2 = 25% of the genes to show the same directional effect of the *crp* ^−^ deletion across all three of them. In fact, 21 of the 25 genes identified as the core CRP regulon show the same directional effect of the *crp* deletion in all three strains, and this pattern is significantly different from the expected 25% based on a binomial test (*p* < 0.0001). We are confident, therefore, that the core regulon includes mostly genes that do, in fact, respond similarly across these strains to the loss of the *crp*-encoded function.

**Table 1:**
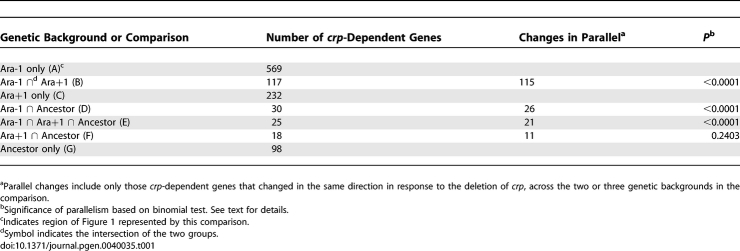
Comparisons of the CRP Regulons Inferred for Evolved and Ancestral Genetic Backgrounds

Region B is of particular interest because it includes those genes that were not identified as belonging to the ancestral CRP regulon, but belong to the regulon in both independently evolved lines. Of the 117 genes in this region, 115 (98%) had the same directional change in both lines with respect to the effects of the *crp* deletion. This parallelism is dramatically different from the 50% correspondence that one would expect if measurement noise had generated spurious associations that defined this region of overlap (*p* < 0.0001). Of the 115 genes that showed parallel changes in the two evolved lines, 78 (68%) had higher expression in association with the functional *crp* ^+^ allele, while 37 were more highly expressed when *crp* was deleted. Therefore, most genes that were recruited in parallel to the CRP regulon have increased reliance on the functional *crp* gene for their expression (binomial test, *p* = 0.0002).

Using the same strategy outlined above, we can be confident that only one of the remaining two regions is biologically meaningful. Regions D and F represent the overlap between the regulons inferred for the ancestor and for the Ara-1 and Ara+1 evolved strains, respectively (Figure 1). Region D shows an excess of genes that change in the same direction; of the 30 genes in this region, 26 (86%) change in parallel (binomial test, *p* < 0.0001, Table 1). By contrast, the number of genes that changed in the same direction in region F was not greater than expected by chance alone (11 of 18, 61%, binomial test, *p* = 0.2403, Table 1). This outcome does not mean that all of the genes in region F necessarily reflect spurious associations with the CRP regulon, but the statistical tests do not support that most of the associations are real based on the criterion of concordant directionality.

It is clear that many genes were recruited to the CRP regulon in the evolved lines. But have other genes lost their dependence on *crp* in the evolved lines? Of the 171 genes identified as the ancestral CRP regulon (regions D, E, F, and G in Figure 1), only 25 were significantly affected by the *crp* deletion in both evolved lines (region E). This discrepancy may indicate that many genes became decoupled from the CRP regulon, but this inference is subject to the problem of false negatives (i.e., failing to confirm a genuine association owing to limited statistical power). We therefore reframed the question to ask whether there was a positive association between those genes that were dropped from the CRP regulon in one evolved line with those that were dropped in the other evolved line. We performed a contingency test using all the genes comprising the ancestral CRP regulon to measure this association. Indeed, there is a highly significant association between loss of a gene from the ancestral regulon in one evolved line and its loss in the other evolved line (Fisher's exact test, *p* < 0.0001). Of the genes in the ancestral regulon that were retained in the CRP regulon by the Ara+1 evolved line (regions E and F), only about 42% (18/43) were dropped by the Ara-1 line. But of those genes in the ancestral regulon that were dropped by Ara+1 (regions D and G), some 77% (98/128) of them were also dropped by Ara-1. We conclude that the parallel recruitment of many genes to the CRP regulon in these evolved lines was accompanied by the parallel loss of other, albeit fewer, genes from the ancestral regulon.

### Nature of Evolved Changes in the CRP Regulon

These analyses indicate that the CRP regulon was substantially altered in the evolved lines relative to its ancestral state, with many parallel changes in the two evolved lines. These changes could have occurred by at least three distinct mechanisms, including any combination thereof. First, both lines could have evolved changes in the pleiotropic action of CRP. These changes could occur through mutations in either *crp* or *cyaA*; the latter gene encodes adenylate cyclase, the enzyme responsible for synthesis of cAMP [49]. cAMP binds to CRP to make the active regulatory complex, cAMP-CRP. A mutation in either of these genes could change the affinity of the cAMP-CRP regulatory complex for target gene operator sequences. To test this possibility, we sequenced the upstream and coding regions of both *crp* and *cyaA* in all three strains. However, we found no mutations in either gene, indicating that the evolved changes to the CRP regulon were not caused by differences in the cAMP-CRP complex itself.

A second possibility is that mutations were substituted in the evolved lines in each gene that was recruited to the CRP regulon so as to bring that gene's expression under the control of *crp*. Other mutations would be required for each gene that dropped out of the ancestral regulon. This hypothesis requires hundreds of mutations in each evolved line, on the order of the number of *crp*-dependent genes that were either recruited to or dropped from the CRP regulon during the evolution experiment. However, both theoretical and empirical evidence indicates that the number of mutations substituted in either of these evolved lines is less than 100 [36,51], and thus much lower than the number of changes to the CRP regulon supported by our analyses (Figure 1; Table 1). Moreover, only some of the mutations that were substituted could be expected to interact with *crp*. Therefore, we consider this explanation to be insufficient to explain our finding of widespread evolved epistatic interactions with *crp*.

A third hypothesis to account for the evolved changes to the CRP regulon is that mutations were substituted in a relatively few other regulatory genes that interact with one or more genes in the existing CRP regulon. The net effect of these few substitutions would be, in effect, to rewire the interacting gene-regulatory networks. Thus, under this model, genes under the control of these intermediaries would come under the influence of *crp* without requiring mutations at each of the affected loci.

To explore the nature of the epistatic interactions between *crp* and the mutations in the evolved strains, we analyzed *crp*-dependent expression changes in the context of the characterized E. coli transcriptional network. The network that we used is based on a comprehensively curated database of interactions (see Materials and Methods). This network comprises 1,217 genes, including 135 regulatory genes that mediate a total of 2,333 transcriptional interactions. If the increase in the number of *crp*-dependent genes was mediated by changes in the interactions between *crp* and other regulatory genes, then we expect the evolved changes to be modular, i.e., the changes in expression should be concentrated in regulons under the control of some of those regulatory genes. To test this prediction, we quantified the expression changes within all the characterized regulons using a previously described method (see Materials and Methods section for details) [52].

As shown in Figure 3, 20 of the 135 regulons we surveyed were significantly differentially regulated by *crp* in at least one of the evolved lines compared to the ancestor (using a double Z-score cutoff criterion of 2.0, which corresponds to *p* ≈ 0.05 [52]). Of these 20 regulons, 14 changed independently in both evolved lines, which is significantly more than expected by chance (Fisher's exact test, *p* < 0.0001). Moreover, all 14 of these regulons were affected in the same direction in both evolved lines (binomial test, *p* < 0.0001). Therefore, at the regulon level, as well as at the level of individual genes, epistatic changes were largely parallel. Twelve regulons – those controlled by *crp*, *dgsA*, *dhaR*, *flhC*, *flhD*, *fliA*, *galS*, *glnL*, *hdfR*, *malT*, *mlc* and *rbsR* – evolved to become less sensitive to the *crp* deletion. Only two regulons, controlled by *lrp* and ppGpp, a small molecule whose cellular concentration is controlled by the *spoT* and *relA* genes***,*** became more sensitive to the *crp* deletion in the evolved lines than in the ancestor.

**Figure 3:**
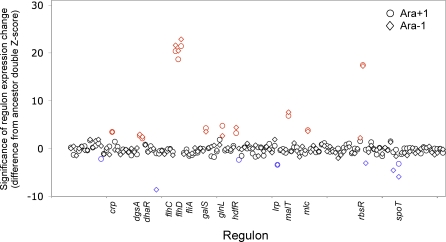
Regulon-Level Analysis of Evolved Changes in *crp*-dependent Gene Expression Regulons include sets of genes that are known to be directly controlled by a particular regulatory gene. The *y*-axis shows the significance level of the change in a regulon's dependence on *crp* in an evolved background relative to the ancestor, where a double Z-score value of 2 corresponds to a *p-*value of ∼0.05. The 135 regulons characterized in E. coli are arranged alphabetically along the *x*-axis, but only those regulons that changed significantly in both evolved lines are listed by name. Blue and red symbols indicate regulons that became significantly more or less sensitive, respectively, to the *crp* deletion in an evolved background. See text for additional information.

Curiously, the numerical imbalance at the regulon level is opposite in direction to our earlier finding that many more genes were recruited in parallel to the CRP regulon by the evolved lines than were dropped from the ancestral regulon. In any case, this regulon-based approach confirms that not all genes were individually recruited to, or dropped from, the CRP regulon, but rather many of them came ‘bundled' in terms of their associations with other regulons, consistent with our third hypothesis. It is also interesting, and consistent with this interpretation, that three of the genes identified as candidates on the basis of this regulon approach have been shown previously to contain beneficial mutations in one (*spoT* [37]) or both (*malT* [40]; and *rbsR* [50]) of the evolved lines.

## Discussion

We deleted *crp*, a major regulatory gene in E. coli, and measured the effect of this deletion on the expression profile of two strains that had evolved independently for 20,000 generations and on their ancestor. The two main effects of deleting *crp* in the evolved and ancestral strains can be summarized as follows. (1) The CRP regulon underwent massive changes during the evolution experiment. These changes reflect a large number of genes that were recruited to the regulon, and a smaller number of genes that were lost from the regulon, during evolution. (2) A striking degree of parallelism was observed in the recruitment of genes to the CRP regulon in the two evolved strains and in the pattern of *crp-*dependent changes across characterized regulons*.* Below, we discuss the significance of these changes to the CRP regulon with respect to an assessment of epistatic interactions between *crp* and mutations fixed during evolution.

### Growth Rate Changes and the Effect of Deleting *crp*

The large number of expression changes caused by the *crp* deletion makes it difficult, if not impossible, to determine the precise physiological basis for the much larger reduction in growth rate of the evolved lines with this deletion. In fact, there may well be multiple factors involved, stemming from the ‘bundling' of regulatory modules that appears to have occurred in the evolved lines as a large number of genes came under the control of *crp*. Here we suggest one possible mechanism, but we do not mean to exclude other possibilities.

More highly connected computational and biological networks are thought to be more robust to the disruptive effects of environmental and genetic perturbations because they buffer against those perturbations [53–56]. Elimination of a network hub may thus reduce the network's capacity to resist perturbations. A recent analysis of the transcriptional connectivity of the E. coli genome predicted that deleting *crp* should substantially reduce that connectivity; on average, 3.96 transcriptional connections separate two randomly chosen genes in the wild-type network, increasing to 4.48 in an otherwise identical *crp* ^−^ network [56]. Experiments with the ancestral strain used here showed that disruptions of hub genes tended to reduce its robustness to environmental perturbations to a greater degree than did disruptions of other genes, although this pattern was not seen for robustness to mutations [56]. Theoretical models predict that beneficial mutations will tend to have deleterious pleiotropic effects, even if they confer a net benefit [57,58]. Therefore, a plausible explanation for the increased growth-rate sensitivity of the evolved lines to the *crp* deletion is that, without a functional *crp* gene, they are less well buffered to the pleiotropic side-effects of the otherwise beneficial mutations that arose during the evolution experiment.

### Identifying CRP Regulon Changes

The individual gene and regulon based approaches we used to characterize the overall evolutionary changes to the CRP regulon gave rather different patterns. Considering the individual gene approach, genes recruited to the CRP regulon in at least one evolved line outnumbered losses in at least one line by 918 to 146 (regions A + B + C vs. D + F + G, Figure 1; Table 1). If new epistatic interactions involving regulatory genes were driving these changes, we expected that, in turn, we would see an overall tendency for regulons to show increased *crp*-dependence in the evolved lines. By contrast, only 7 regulons showed significantly increased *crp*-dependence in one or both evolved lines compared to 13 with reduced dependence (Figure 3). Regulons are simply groups of co-regulated genes, so how can it be that more genes, but fewer regulons, became more dependent on *crp* during evolution? We consider three explanations for this apparent discrepancy.

First, the fraction of *crp*-dependent genes that were recruited to the CRP regulon could differ between the subset of genes included in the regulon analysis and the complete set of genes. If so, changes in regulon-level *crp*-dependence would not be predictable from the overall pattern of gene recruitment and loss. To address this possibility, we repeated our earlier analysis, comparing the number of genes recruited to, and lost from, the CRP regulon during evolution, but considering only those genes that were included in the regulon analysis (∼28% of total genes). We found that the proportion of genes recruited to the CRP regulon was indeed lower in this subset (Fisher's exact test, *p* = 0.0009), but the number of genes recruited was still several-fold higher than the number that were lost (241 gains, 67 losses). Thus, this effect alone does not alter the expectation that an evolved increase in the number of *crp*-dependent genes should also increase the number of *crp*-dependent regulons.

Second, the regulon analysis is limited to previously characterized regulons. Several mutations substituted during the evolution of the Ara-1 and Ara+1 lines have been shown to affect the physical topology, or supercoiling, of DNA [59]. Such changes are known to influence the accessibility of regulatory protein binding sites, which can thereby substantially alter the range of genes available to be regulated [60]. Indeed, one previous study has shown that there are a number of low-affinity CRP binding sites in the E. coli chromosome that can be influenced by topological changes [61]. If evolved topological changes altered the identity of genes that comprise a known regulon, it would make it harder to identify evolved changes in that regulon, because the actual set of co-regulated genes would have diverged from the presumptive regulon that is being tested.

Third, the particular statistical method we used to assess the significance of expression change within regulons may itself be more sensitive to detecting loss rather than gain of *crp*-dependence in the evolved populations relative to the ancestor. The test compares the effect of deleting *crp* on gene expression within a regulon to a baseline covariance calculated by repeatedly sampling the same number of random genes (chosen from among the 1217 genes included in our transcription regulation network). However, deleting *crp* had a much greater effect on the overall expression profiles of the evolved lines than on the ancestor's profile; thus, the distribution of covariances was broader in the evolved lines, making it more difficult to detect changes in regulon *crp*-dependence. Even so, we could identify significant changes in three regulons with known mutations in the evolved lines, which indicates that this analysis generated a biologically meaningful signal despite this constraint on statistical resolution.

It is also difficult to know how many of the changes to the CRP regulon that occurred during the evolution of the Ara-1 and Ara+1 lines indicate new or altered molecular interactions among regulatory genes and their products, and how many reflect indirect effects of physiological changes on gene expression. In particular, it is certainly possible that some of the expression changes reflect the growth-rate differences between strains, especially given the more severe growth-rate impairment caused by the *crp* deletion in the evolved lines. One potential approach to test for growth-rate effects would be to compare the gene-expression profiles in these strains when they are all forced to grow at the same rate, which could be achieved by growing them in separate but identical chemostat vessels, where the dilution rate is slow enough to allow each strain to sustain itself and reach steady-state [62]. In any case, the fact that differences in growth rate may mediate some of the altered interactions demonstrated by our experiments does not alter the conclusion that these changes reflect widespread epistasis between *crp* and the different genetic backgrounds. In other words, it may be misguided to take a complex web of genes and their interactions, which collectively determine growth rate, and then attempt to remove the effect of growth rate in order to say that the genes do not interact.

### Genetic Changes Underlying Changes to the CRP Regulon

A key advantage of the regulon approach that we used is that it can identify specific genes that may mediate the new epistatic interactions. The overlap in the identity of the regulons affected by the deletion of *crp* marks their regulators as candidates for having substituted mutations in the evolved lines that interact epistatically with *crp*. Indeed, of the 14 regulatory genes controlling the regulons identified as having changed significantly in *crp*-dependence in both evolved genotypes, three of them – *spoT*, *malT* and *rbsR* – have been shown to contain beneficial mutations [37,40,50]. Two of these genes control well-characterized regulons: *rbsR* regulates the expression of six genes (including itself) that control ribose catabolism, and *malT* encodes a positive regulator of ten genes in two operons that control maltose catabolism. A cAMP-CRP binding site is located upstream of both of these regulatory genes [63]. The mutations in *rbsR* involve deletions of this gene, and the majority of the *rbs* operon, in both evolved lines, such that the operon is no longer induced by the cAMP-CRP complex in the low-glucose environment in which the cells were grown, explaining why the regulon became less responsive to the *crp* deletion. The mutations in *malT* produce amino-acid substitutions in the coding region of this gene in both evolved lines. Although the biochemical consequences of these mutations have not yet been determined, our results are consistent with previous findings that they reduce expression of the *mal* operons, making them less responsive to cAMP-CRP [40].

The underlying basis of the epistasis between *spoT* and *crp* is less clear; the *spoT* regulon is quite large and not as well characterized as the *rbsR* and *malT* regulons. The *spoT* gene influences cAMP-CRP activity by changing the intra-cellular concentration of ppGpp, thus providing a mechanism by which a mutation in this gene could affect the composition of the CRP-regulon [64]. However, there is no known reciprocal link between CRP-cAMP activity and ppGpp. Therefore, it is not clear how a mutation in *spoT* would change the dependence of the *spoT* regulon on *crp*. In future work, we will sequence regulatory genes in the 11 other regulons that changed their *crp*-dependence in both evolved lines, to determine whether they also contain mutations that might explain their altered interactions with *crp*.

In this study, we deliberately used a mutation of large effect – a deletion of *crp* – to examine as fully as possible the evolved changes to the CRP regulon. However, it is also interesting to consider whether such widespread changes would result from ‘smaller' disruptions such as point mutations. Although it seems reasonable to imagine that the average effect of point mutations would be smaller, it is important to remember that the evolved epistatic interactions revealed by deleting *crp* were caused by mutations in other genes. We do not yet know the identity of all these mutations, but those that we have discovered include a mixture of point mutations, deletions, and insertions [37,39,40,50]. Targeted reversion of these mutations, including even ‘small' point mutations, are likely to have an effect on the extent of the evolved epistatic interactions.

Two previous studies examining epistasis in the context of this same evolution experiment compared the effects of a set of 12 transposon-insertion mutations on the fitness of the ancestor and clones isolated from an evolved line at 1,000 and 10,000 generations [28,29]. Epistatic interactions between an insertion mutation and evolved mutations elsewhere in the genome were inferred when the effect of the insertion mutation on fitness was significantly different in the ancestral and evolved backgrounds. The ability of that approach to detect epistasis is limited by the resolution of fitness measurements. Moreover, fitness integrates effects over all underlying traits, such that epistatic interactions at lower levels may be obscured. The approach used here overcomes this limitation by treating the expression level of each gene as a phenotypic trait. The large number of such traits that can be surveyed simultaneously and with considerable precision may allow the detection of more subtle differences in the effect of a mutation in different genetic backgrounds, and this approach may thus provide more sensitive detection of epistatic interactions. Furthermore, the intimate relationship between genotype and expression levels offers greater opportunity for mechanistic insights into the nature of the epistatic interactions thus discovered.

In conclusion, we have used expression arrays to analyze the extent of epistatic interactions occurring between *crp* and mutations that fixed during 20,000 generations of evolution in *E. coli*. These epistatic interactions are widespread, and many of them emerged in parallel in independently evolving lines, indicating that the derived mutations – and perhaps also the altered web of interactions – contribute to the bacteria's adaptation to the experimental environment. Indeed, some of the parallel changes in epistatic interactions involve known regulons in which key regulatory genes have been shown to harbor beneficial mutations in the evolved lines. Finally, the use of expression profiles to detect epistatic interactions has the advantage that it does not require *a priori* knowledge of the molecular and physiological bases of interactions that are discovered, although this approach can be integrated with such knowledge to inform our understanding of genotypic and phenotypic variation.

## Materials and Methods

### Strain histories.

Twelve lines of E. coli B were founded from two ancestral types differing only by a selectively neutral marker. These lines were propagated at 37 °C in a glucose-limited minimal medium for 20,000 cell generations [35,36]. Expression profiles were obtained from *crp^+^* clones isolated from two of these lines (designated Ara-1 and Ara+1) after 20,000 generations of evolution and from the ancestor to one of those lines. These profiles were compared to otherwise isogenic *crp* ^−^ derivatives of these same three clones, constructed as described below. Only one ancestral type was examined here because no difference was detected between the expression profiles of the Ara- and Ara+ ancestors in a previous study [37]. During the evolution experiment, four of the twelve lines evolved mutator phenotypes; however, the mutation rate of both lines used in this study remained at the low ancestral level [51,65].

### Strain construction.

The *crp* (*c*AMP *r*eceptor *p*rotein) gene was deleted from the ancestral and evolved strains using a suicide plasmid-mediated approach described previously [50,66]. Briefly, a 1,016-bp PCR product containing a 591-bp in-frame *crp* deletion allele was cloned into pDS132, a derivative of pCVD442 [50,66,67]. This plasmid was transferred by conjugation into recipient cells, in which the deletion would be introduced, and chloramphenicol-resistant cells (resulting from chromosomal integration of the non-replicating plasmid) were selected. Resistant cells were streaked onto LB + sucrose agar to select cells from which the plasmid had been lost through intra-chromosomal recombination (pDS132 encodes the *sacB* gene conferring susceptibility to sucrose). These cells were then screened for the presence of the *crp* deletion allele by PCR and hybridization experiments. Several independent *crp* ^−^ strains were made in each background. All replicate *crp* ^−^ strains exhibited the same characteristic change in colony morphology relative to their respective *crp^+^* progenitors. The *crp* ^−^ strains used in this study were further verified by sequencing the *crp* deletion allele and surrounding regions. This sequencing confirmed the deletion allele and ruled out the possible introduction of secondary mutations in the flanking regions of the introduced allele.

### Growth rate assays.

Prior to growth rate assays, all strains were grown for one day under the same conditions that prevailed during the evolution experiment, in order to ensure they were similarly acclimated to the culture conditions (37 °C, minimal medium supplemented with 25 mg/L glucose). Following this pre-incubation, strains were diluted 1:100 into 300 μL of fresh media contained in a well of a standard 96-well microtiter plate. This plate was incubated with periodic shaking at 37 °C and the change in optical density at 450 nm (OD_450_) was monitored using a Versamax Pro spectrophotometer. The maximal growth rate of each genotype was estimated by calculating the slope of the natural log of OD_450_ against time during the period of most rapid growth. Reported growth rates are the average of 10 independent estimates for each strain.

### RNA isolation and macroarrays.

Expression profiles were generated using Panorama E. coli cDNA macroarray membranes (Sigma-Genosys). Prior to RNA extraction, all strains were acclimated to the same conditions used during the evolution experiment, then diluted 1:100 into fresh medium and grown to mid-exponential phase. Cells were harvested using 0.45 μm filter units (Nalgene) and re-suspended in a 1:1 mix of buffer and RNAlater RNA stabilizing solution (Ambion). RNA was obtained using the Qiagen RNeasy system, including an additional step to remove contaminating genomic DNA using the Qiagen on-column DNAse kit. Subsequent cDNA production, labeling, and hybridization were performed according to the instructions of the macroarray membrane manufacturer. Following hybridization, membranes were washed as per manufacturer instructions and exposed to Kodak PhosphorImager screens for 24 h. Exposed screens were scanned on a STORM 840 PhosphorImager (Molecular Dynamics). Image files were analyzed using Arrayvision software (Version 6.0, Imaging Research), and the output exported to Microsoft Excel for manipulation. Three independent mRNA preparations and hybridizations were performed for each strain. All RNA isolation and hybridization steps were done in complete blocks to control for any effects of day-to-day variation on results.

### Statistical analyses.

The Panorama macroarray consists of 4,290 PCR products, each corresponding to an individual open reading frame from E. coli K-12, spotted in duplicate onto a nylon membrane. To measure the expression level of each gene, we subtracted the average background from the mean of the two readings to calculate its adjusted expression level. To standardize expression and thereby account for possible differences between arrays, the adjusted expression levels were normalized to the median gene expression value obtained for the array. Standardized values were log_10_-transformed, and paired *t*-tests were performed using the transformed values from replicate arrays to identify genes whose expression had changed significantly. Expression values obtained from RNA samples isolated on the same day for *crp* ^+^ and *crp* ^−^ derivatives of each strain were paired in this analysis. These tests were performed using the paired-data analysis option of the web-based Bayesian statistical program Cyber-T (http://cybert.microarray.ics.uci.edu/) [68,69]. A sliding window of 201 genes and a confidence limit of 2 were used in this analysis. This program reduces the number of false-positive differences expected in a comparison of replicate expression profiles from two strains through the use of Bayesian estimates of variance among replicate gene measurements. The use of a formal statistical test avoids arbitrary cut-offs, but it is still expected to identify many false-positives. To account for this, additional tests were performed on subsets of the data, as described in the text, using Microsoft Excel and the SAS frequency procedure (SAS Institute V8, 2000). These tests sought to compare observed distributions of *crp*-dependence to those expected by chance alone. Binomial tests were used to test the directionality of *crp*-dependent gene expression changes, reflecting our null hypothesis that genes identified by chance as being significantly changed in two genetic backgrounds would be equally likely to change in the same or in different directions. We also performed Fisher's exact tests to compare the numbers of genes changed in each direction to expectations given observed inequalities in the number of up- and down-regulated genes in the corresponding CRP regulons. In all cases, the outcomes of these Fisher's exact tests were qualitatively consistent with the reported Binomial tests (data not shown).

### Regulon analysis.

A transcription interaction network for E. coli was compiled from regulatory interactions downloaded from regulonDB [63] (data were accessed and downloaded on June 22, 2006), and from relevant data presented elsewhere [70,71]. We define regulons as groups of genes directly regulated by the regulatory genes present in these datasets. Regulatory genes were defined as genes with outgoing links to at least two other genes. Because our aim was to increase the sensitivity of measuring regulatory changes caused by altered activity of any regulatory gene, those genes that were co-regulated within operons were considered as having separate links to any gene that controlled the transcription of that operon. The regulatory genes *spoT* and *relA* were considered together because they combine to control the level of (p)ppGpp, a key regulatory molecule that binds to RNA polymerase and alters its affinity for transcriptional start sites [72,73]. Although *spoT* and *relA* control the same set of genes through their action of modulating levels of (p)ppGpp, they do so in response to different environmental conditions [74]. Moreover, they are involved in distinct sets of protein-protein interactions [75,76]. Therefore, despite the apparent regulatory redundancy, we expect that a mutation altering the regulatory capacity of either gene would be discernable through changes in the expression of co-regulated genes. In all, our transcription regulation dataset comprised 2,333 interactions and included 1217 of the 4290 genes assayed on the Panorama macroarray.

To measure the extent and significance of *crp*-dependent expression change within the 135 regulons identified in this dataset, we used an algorithm described by Balazsi et al*.* [52]. This algorithm calculates a double-Z score for each regulon based on the covariance of changes in expression caused by the *crp* deletion among genes in the regulon relative to an average background covariance calculated over 1,000 samples of an equal number of randomly chosen genes. This score therefore provides a measure of expression change within a regulon that controls for differences between paired *crp* ^+^ and *crp* ^−^ strains in their overall measurement variability.

We also considered more extensive interaction groups that included indirectly regulated genes (origons in the terminology of ref. 52). Results obtained using these groups were qualitatively similar to those obtained in the regulon analysis (data not shown).
